# Methods of Protection of Electrochemical Sensors against Biofouling in Cell Culture Applications

**DOI:** 10.1021/acsomega.3c07660

**Published:** 2024-01-18

**Authors:** Elżbieta Jarosińska, Zuzanna Zambrowska, Emilia Witkowska Nery

**Affiliations:** Institute of Physical Chemistry, Polish Academy of Sciences, Warsaw, ul. Kasprzaka 44/52, 01-224 Warsaw, Poland

## Abstract

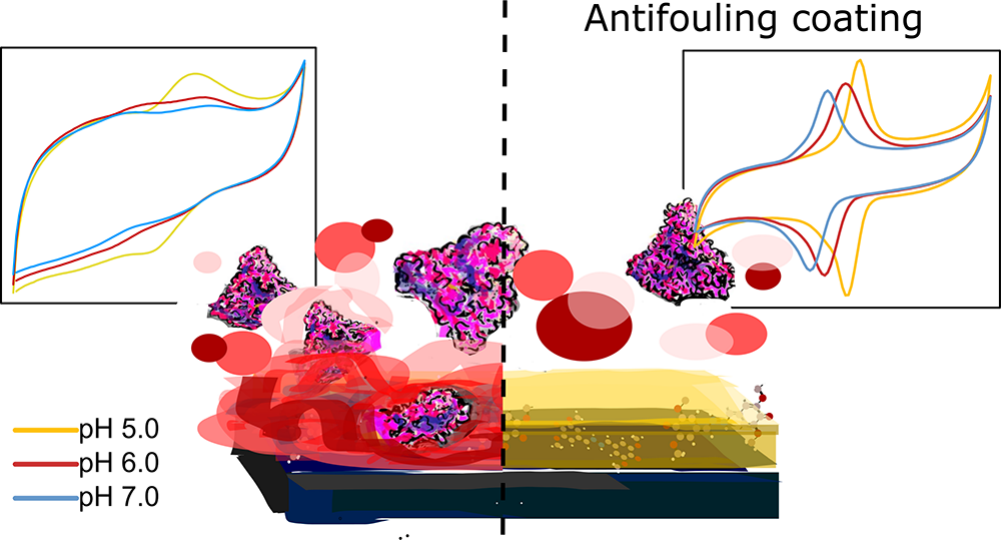

In this work, we evaluated more than 10 antifouling layers presenting different modes of action for application in electrochemical sensors. These layers included porous materials, permselective membranes, hydrogels, silicate sol–gels, proteins, and sp^3^ hybridized carbon. To evaluate the protective effects of the antifouling modification as well as its impact on the catalyst, we adsorbed a redox mediator on the electrode surface. Five of the tested coatings allowed us to preserve the electrochemical properties of the tested mediator. Later studies showed that sol–gel silicate layer, poly-l-lactic acid, and poly(l-lysine)-*g*-poly(ethylene glycol) were the only ones capable of sustaining the catalyst’s performance during prolonged incubation in a cell culture medium. The highest signal deterioration was observed, as expected during the first few hours of incubation in a cell culture environment. Tested layers exhibited different dynamics of the protective effect. The poly-l-lactic acid layer presented lower changes in the first hours of the study but suffered complete signal deterioration after 72 h. Whereas the signal intensity of the silicate layer was lowered by half after just 3 h but was still visible after 6 weeks of constant incubation of the electrode in the cell culture.

## Introduction

(Bio)fouling is the nonspecific adsorption of molecules at the liquid–solid interface. While the mechanism itself may be different, the problem of biofouling is common for environmental applications and medical devices during extended contact with body fluids, including drug delivery systems and diagnostic devices.^1^

Nowadays, electrochemical sensors have gained great attention due to the possibility of multiplexed determination of certain biomarkers and trace amounts of heavy metals. However, conducting experiments in biological matrices is still a considerable challenge. Most biological samples are a complex mixture of proteins, amino acids, peptides, lipids, and carbohydrates,^2^ which easily adsorb on the probe’s sensing area decreasing long-term stability.

Contaminants add up to finally create an impermeable layer on the electrode surface, significantly affecting the analytical characteristics of the electrochemical sensor and finally leading to loss of sensitivity and reproducibility. The target analyte is usually in a low concentration, and every minor degradation of the sensing area could be disastrous for sensor function. Increased background noise during electrode fouling can completely screen the already extremely low signal of the analyte.^3^

This study aimed to find a method that would protect electrochemical sensors from detrimental effects of cell medium and allow long-term, up to a few weeks, studies of cell cultures. The types of fouling agents are remarkably broad, equally to strategies that have been used to reduce biofouling; therefore, it is crucial to find a specific one for each application. Organic solvents or surfactants may be added to the sample to increase the solubility of reaction products, which coat the electrode surface.^4^ However, this approach is only valid for *ex situ* analysis, as such additives could harm the cells and influence the bioassay.

Apart from additives, electrochemical measurement itself can be used for desorption of species. In electrochemical activation, a single use of cathodic/anodic potential or a train of pulses may reduce adsorption of fouling agents or remove already attached substances.^5^ Electrochemical cleaning can be used to remove adsorbed species through forced oxygen and hydrogen evolution reactions and creation of gas bubbles^6^ or etching of the surface in the case of carbon fiber microelectrodes.^7^ Unfortunately, such an action can be detrimental in the case of surfaces modified with chemical or biological catalysts, resulting in their degradation or physical detachment.

Most antifouling strategies rely on the application of a coating (layer), which can act as a passive barrier by preventing a contaminant from reaching the electrode surface or an active one by releasing some neutralizing agents. Protective layers can modify the surface in a way to minimize interactions with potentially detrimental substances, e.g., by changing the hydrophobicity/hydrophilicity, imparting a charge, etc. Such layers can be formed from polymers that form a physical barrier, preventing access to unwanted species. They can render the surface superhydrophobic, impairing the adhesion of new species. Hydrophobic and uncharged hydrogels prevent fouling not only through the barrier effect but also thanks to strong repulsive hydration forces of the bound water.^8^ Most approaches toward electrochemical sensors use chemical modification as an antifouling coating. A huge advantage of this approach is the wide range of polymers and end-group functionalizations, which can be easily applied to the biosensor surface. Poly(ethylene glycol) (PEG) and its derivatives (e.g., OEG) are one of the common antifouling materials widely used in biomedical applications. PEG layers are nontoxic and biocompatible and can be easily attached to the electrode surface. Additionally, various lengths of their chains result in different thicknesses of monolayers.^9^

Sol–gels are more porous and present higher mechanical and thermal stabilities than hydrogels, which allows for their application in implantable sensors. Porous layers can form the external part of the electrode modification and act as a diffusion barrier, limiting access to only the smallest analytes. In the case of commercial applications, defined and specific parameters of the pore size and their distribution are needed. Polymeric or silica-based membranes are a method of choice in this case due to biocompatibility, stability, and easiness of application.^10^

A promising strategy for preventing infections in the case of point-of-care devices and implantable medical devices is an antimicrobial coating based on drug-releasing material (e.g., antibiotic/chlorhexidine/silver/nitric oxide-releasing coatings).^11,12^ Such active coatings are able to kill microbes that approach the surface of the device; however, applying them as an antifouling layer in cell cultures could influence the performed assays. A coating that does not release any molecules but is based on repellent properties such as cationic biocidal polymers^13^ is more suitable for cell culture application. Photoactive coatings based on metal-oxide nanoparticles gained a lot of attention some time back.^14^ However, the core of the bactericide function that is generation of reactive oxygen species (ROS) is highly detrimental for organic molecules, leading to protein dysfunction, membrane damage, or oxidative stress, which is and could negatively influence the cultured cells.^15^

Instead of using continuous layers, some strategies take advantage of more diffuse coatings. The sensor surface is blocked with a known protein to, in a controlled manner, account for the most drastic drop in sensitivity occurring in the first few hours of contact. In recent years, zwitterionic molecules have also gained increasing attention due to their high oxidative resistance and hydrolytic stability^16,17^ as well as thiolated self-assembled monolayers (SAMs).^18,19^ Carbon materials can also be used in this way, but, in this case, the protective effect is provided through sp^3^ hybridization and increased surface area. The nanoengineered surfaces, widely investigated in many laboratories, provide extremely high electroactive surface, increasing sensitivity and level of electrochemical signals. Due to the irregular morphology, they are able to obtain lower LOD in comparison to planar carbon electrodes.^20^

Many antifouling strategies have been presented earlier. The objective of this study was to identify a surface modification that would not have an impact on the electrochemical properties of various sensors to be used in the future. The selected method must not damage the catalyst, affect the reaction mechanism, or alter the reaction environment, such as through localized pH changes. Instead of an external redox probe added to the solution^21^ as in the case of (ruthenium II/III hexaammine) redox couple our work is based on an internal redox mediator adsorbed on the electrode surface. Syringaldazine was utilized as a model catalyst due to its easy adsorption onto carbon surfaces and its simple response to pH changes, as described by Michalak et al.^22^ Although the modification is very stable in buffer solutions, it rapidly deteriorates in more complex media, making it an excellent tool for measuring the advantageous effects of the antifouling layers. This approach was used to screen more than 10 different antifouling layers and evaluate both the protective abilities as well as the impact on the catalyst, which was not possible with an external mediator.

## Experimental Section

### Fabrication of Electrodes

Pentel Ain Stein 2B 0.2 (made in Japan, Pentel Co.Ltd.) pencil lead was positioned in a 1.6 mm diameter glass capillary and fixed in place by heating one end of the capillary with a Bunsen burner. A copper wire was inserted in the other end, glued together with the pencil by means of conductive silver glue (16062 Pelco Conductive Silver Paint, Ted Pella), and additionally fixed with hot glue to form a stable electrode connection. The electrodes were first polished on sandpaper and later on a piece of copy paper. After initial screening was performed by running a cyclic voltammogram in a redox probe solution, electrodes that showed a similar current range indicating proper enclosure of the pencil rod in glass were further polished using an alumina slurry.

### Modification of the Electrodes with Syringaldazine

All carbon electrodes (glassy carbon [eDAQ], Screen printed electrodes [AC10.W4.R2 BVT Technologies, a.s.], pencil electrodes) were modified by immersion in a 0.5 mg/mL solution of syringaldazine (99%, Sigma-Aldrich) in ethanol (99.8%, POCH) for 60 s and dried under ambient conditions, according to reference.^22^

### Electrochemical Measurements

After modification with syringaldazine electrodes was tested in buffers of different pH values. First tests were performed in buffers from pH 4 to 9.5 (acetate, phosphate, and carbonate-bicarbonate buffers), but the most basic solution sometimes had a detrimental effect on the modifications. To keep a similar composition to the test solution, phosphate buffer of three different pH values was chosen for subsequent tests of the electrodes. All electrochemical measurements were performed with a PalmSens 4 potentiostat in a three-electrode system consisting of a selected carbon working electrode, Ag/AgCl (3 M KCl) reference electrode (IJ Cambria Scientific Ltd.), and platinum wire of 1 mm diameter (Mennica Metale Sp. z o.o.) as an auxiliary electrode. If not stated otherwise, cyclic voltammetry (CV) measurements were conducted in the potential range from −0.2 to +0.8 V with 100 mV/s scan rate and 10 mV potential step. Differential pulse voltammetry (DPV) measurements were performed in the potential range from −0.5 to +0.5 V with a 25 mV/s scan rate, 10 mV potential step, 0.2 V potential pulse, and 0.02 ms pulse time. Square wave voltammetry (SWV) measurements were performed in the potential range from +0.8 to −0.4 V with a 10 mV potential step, 100 mV amplitude, and 20 Hz frequency.

### Preparation of Antifouling Layers

Eleven different electrode modifications were tested, some additionally optimized during the experiments.

Methanol (>99.8%), dichloromethane (DCM) (pure p.a.), Nafion, 2-nitrophenyl octyl ether (>99%), o-phenylenediamine (>98%), HEPES (>99.5%), potassium hydroxide (>99,99%) poly-l-lysine hydrobromide, poly(vinyl chloride) (PVC), monomethoxy PEG-nitrophenyl carbonate, PEG diglycidyl ether (PEGDE), and bovine serum albumin (BSA) were purchased from Sigma-Aldrich. Tetrahydrofuran (>99.8%), hydrochloric acid (pure p.a) was from POCH, tetramethoxysilane (TMOS), trimethoxysilylpropyl-*N*,*N*,*N*-trimethylammonium chloride (TMA) (>98%), were obtained from Sigma-Aldrich. Hydroxyethyl methacrylate (HMMA) was from ABCR. Ammonium bicarbonate (pure p.a) was from Chempur and PBS tablets 7.4 pH were from ROTH. Water was filtered and deionized with a Sartorius Arium Comfort I system.

#### Nafion

5 μL of Nafion was drop-cast on the electrode surface and left to dry or the electrode was dipped three times in a 5% solution of Nafion and dried in 120 °C for 30 min.^21,23,24^

#### Polyorthophenylenediamine

Electrodes were placed in 300 mM o-phenylenediamine (OPD) solution in degassed PBS pH 7.4 buffer, and 700 mV vs Ag/AgCl 3 M KCl was applied for 15 min.^25^

#### Polyvinyl Chloride

33 mg of PVC and 66 mg of plasticizer 2-nitrophenyl octyl ether were mixed with 500 μL of tetrahydrofuran. The polymer solution was drop-cast on the electrode surface and left to dry. The polymer:plasticizer ratio was chosen based on the composition of ion-selective electrodes.^26^

#### Poly-l-Lactic Acid

1 g of PLLA was dissolved in 3 mL of dichloromethane using an ultrasonic bath. Next, 1 g of ammonium bicarbonate was added. The solution was drop-cast on the electrode surface, and after 5 min the electrode was dipped in 85 °C for 5 min to initiate pore formation and in cold water for 20 min to quench the reaction. Electrodes were left to dry at room temperature.^27^

#### Poly(Ethylene Glycol) Diglycidyl Ether

100 mg of PEGDE was mixed with 990 μL of distilled water and 10 μL glycerol. After mixing, the solution was drop-cast on the surface of the electrodes. The electrodes were left in the oven for 2 h at 55 °C.^28^

#### Hydroxyethyl Methacrylate

HMMA was mixed with *N*-vinyl-2-pyrrolidinone in a 3:2 molar ratio. Argon was used to deoxygenate the solution. Next 50 μL of Benacure 1173 (2-hydroxy-2-methyl-1-phenyl-1-propanone was added. After drop-casting of the final solution, electrodes were positioned under a UV lamp (365 nm) and irradiated for 5 min.^29^

#### Tetramethoxysilane (TMOS) Silicate Matrix

74 μL of methanol was mixed with 11 μL of 0.01 M HCl, 30 μL of distilled water, and 105 μL of TMOS, using a vortex after each addition. The solution was drop-cast on the electrode surface. Electrodes were left to dry and used on the next day.^30^

#### TMA and Tetramethoxysilane Silicate Matrix (TMA/TMOS)

270 μL of TMOS with 30 μL of TMA, 75 μL of distilled water, 185 μL of methanol, and 5 μL of 11 M aqueous HCl were mixed and drop-casted on the electrode surface. Electrodes were left to dry and used on the next day.^31^

#### Bovine Serum Albumin

BSA was deposited directly or mixed in the PEGDE hydrogel. In the case of PEGDE, 150 mg of BSA was mixed with 100 mg of PEGDE, 990 μL of distilled water, and 10 μL glycerol. After mixing, the solution was drop-cast on the surface of the electrode. Electrodes were left in the oven for 2 h at 55 °C. For the direct deposition, electrodes were dipped in 100 mg/mL BSA solution for 2 h.

#### Poly(l-Lysine)-g-Poly(Ethylene Glycol)

5 mg of poly-l-lysinehydrobromide was dissolved in 0.1 mL of 50 mM sodium borate buffer (SBB), pH 8.5. 0.2 g monomethoxy PEG-nitrophenyl carbonate was added to the dissolved PLL. The reaction was allowed to proceed for 6 h at room temperature, after which the reaction mixture was filtered (0.45 μm pore size filter).^32^

#### Nanodiamond

10 μL of TMA/TMOS solution prepared as before was added to 1 mL of water containing 3 mg of nanodiamond. After vortexing, the solution was drop-casted on the electrode surface. Electrodes were left to dry overnight.

### Routine Cell Culture

HeLa cells from the American Type Culture Collection (ATCC, Manassas, USA) were cultured as a standard monolayer in the complete growth medium, supplemented with fetal bovine serum (FBS, Gibco), l-glutamine 1% v/v (Sigma-Aldrich), and the antibiotics: streptomycin [10,000 U ml^–1^] and penicillin [10 mg mL^–1^] 1% v/v (Sigma-Aldrich). Cultures were performed under the standard conditions (37 °C, 5% CO_2_). HeLa cell lines were cultured in Dulbecco’s Modified Eagle’s Medium (DMEM) with low glucose content (Institute of Immunology and Experimental Technology, Wrocław, Poland). Using regular passages, cells were maintained in a logarithmic growth phase. To detach cells from the surface, 0.25% Trypsin–EDTA solution (Sigma-Aldrich) was used.

### AlamarBlue Assay

The dye was applied according to the manufacturer’s protocol (General Method for Measuring Cytotoxicity or Proliferation Using alamarBlue by Fluorescence, Bio-Rad). Five controls were performed: (a) cells with alamarBlue (positive control), (b) cells with 1% Triton-X 100 (negative control), (c) medium alone with dye (blank), (d) dead cells with alamarBlue, and (e) a pure medium without dye. 20–30 μL of specific compounds of layer were dropped on each well and incubated at 37 °C for 48 h. After this time, cells were seeded into a 96-well plate (Greiner Bio-One) with approximately 7500 cells number per well (controlled with Countess II Cell Counter) and were incubated at 37 °C for 24 h. Plates were read after incubation with 10% alamarBlue dye at 37 °C in phenol red-free culture medium (to eliminate the background fluorescence), lasting 4 h. The fluorescence was measured at 590 nm (590/20 filter) for excitation at 560 nm (560/20 filter). A plot of cell viability was made by taking the averages of the six repeats.

### Electrochemical Measurement in Cell Culture

For electrochemical measurements, cells were grown on tissue culture dishes (SARSTEDT, TC Dish 35, ref 83.3900) on which the above-described pencil electrode has been installed. To properly position the working electrode, a small orifice was melted out in the side of the culture dish using a hot metal rod, and the electrode was positioned inside and held in place using hot glue. Regular passages were made in which cells were maintained in a logarithmic growth phase. The working electrode was in the cell culture without interruptions during the whole time frame of the experiment. The reference and counter electrodes were positioned in the cell culture medium from the top for the individual measurements.

## Results and Discussion

### Choice of Electrodes

This study is based on facile modification of carbon surfaces with syringaldazine, which can be later easily monitored electrochemically. As the modification is very stable in buffer solutions but the compound tends to detach in more complex media (e.g., cell medium), we thought it would be an excellent probe to check different protective layers. To take advantage of this fast, one-step modification, electrodes through adsorption from ethanolic solution should be made from carbon.

The first experiments were conducted on standard commercial glassy carbon electrodes 3 mm in diameter (Figure S1). However, we have noted that in some cases such a big area resulted in uneven spreading of the layer, formation of cracks, or adhesion problems in some of the tested materials. As the goal of this work was to find a suitable electrode modification strategy that would be used in electrode arrays in which the size of the electrode ranges from tens to hundreds of micrometers, we decided to use other electrodes with a smaller surface area that could be easily cleaned or bought/fabricated in large quantities, and their surface would be made from carbon to allow for easy modification with syringaldazine.

Commercial, multichannel screen-printed carbon electrodes were our first choice, but after modification with syringaldazine, additional peaks were visible on the voltammograms, and the results were not reproducible (Figure S1(right)). The electrodes were tested in buffer solutions and solutions of standard redox probes in which their response was as expected. We tried to clean the electrodes by washing them in organic solvents or electrochemical cycling in solutions of bases or acids, but it was still not possible to observe a clean signal from syringaldazine. The problem was attributed to inherent impurities from the electroactive paint, which in some way interact with syringaldazine. Such impurities are common in SPE carbon inks and can show up as additional peaks visible in background scans but are usually dwarfed by the signal of the analyte.^33^

**Figure 1:**
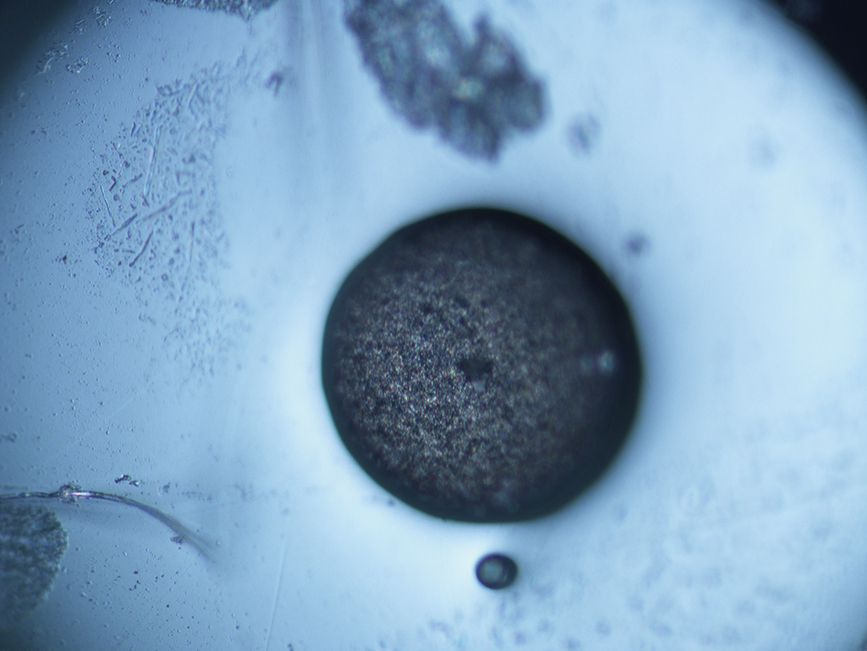
Microscope image of pencil lead electrodes (×10 magnification).

We have decided to use pencil lead electrodes, which are low cost and easy to prepare in large quantities, allowing many tests to be performed simultaneously. Besides low-cost sensing applications, pencil lead electrodes can be used as easily available, disposable sensors from high-quality graphite.^34^ Softer leads (2 or even 8B) with higher graphite to filler content should be used for electrochemical applications.^35,36^ Graphite’s sp2 hybridization should also allow for efficient modification with syringaldazine. Such electrodes can be easily fabricated by embedding the graphite rod from an automatic pencil in an insulating polymer, enclosing it in a micropipette tip, or as was done in this work in a glass capillary (Figure 1).

### Evaluation of the Protective Electrode Modification

**Figure 2:**
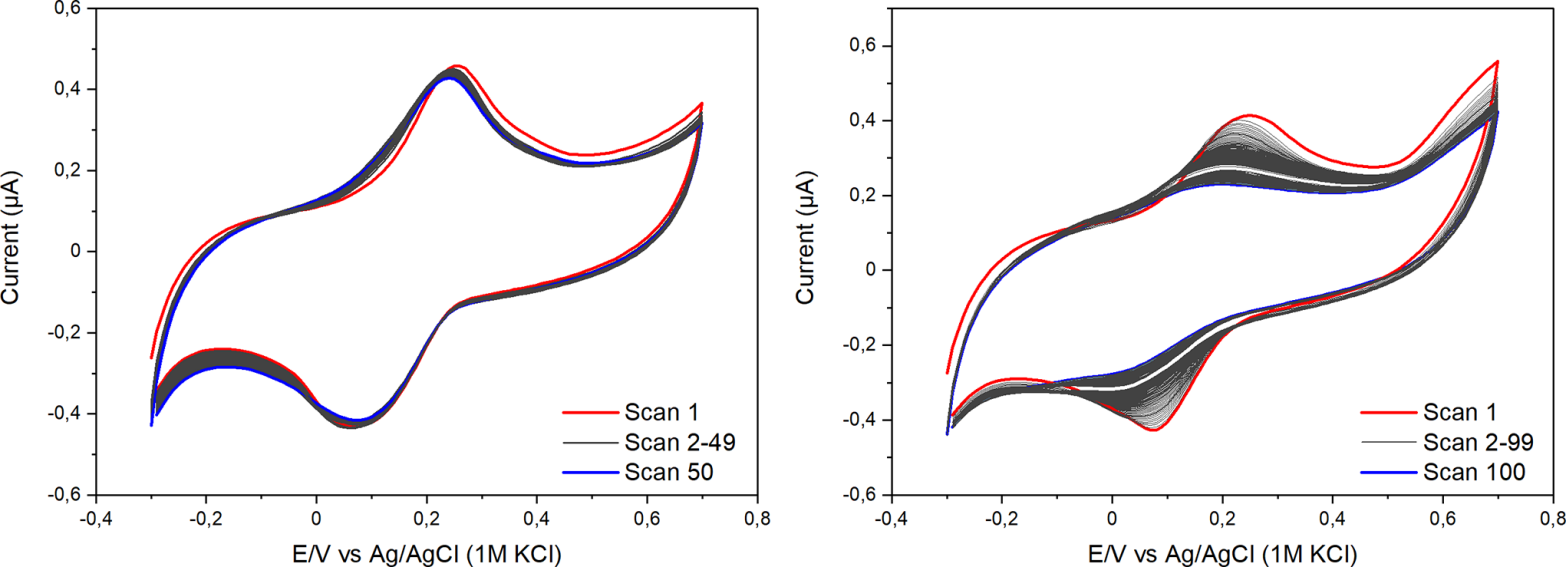
Continuous scanning of a pencil graphite electrode covered with syringaldazine. Left: in buffer solution; and right: in cell culture medium.

**Figure 3:**
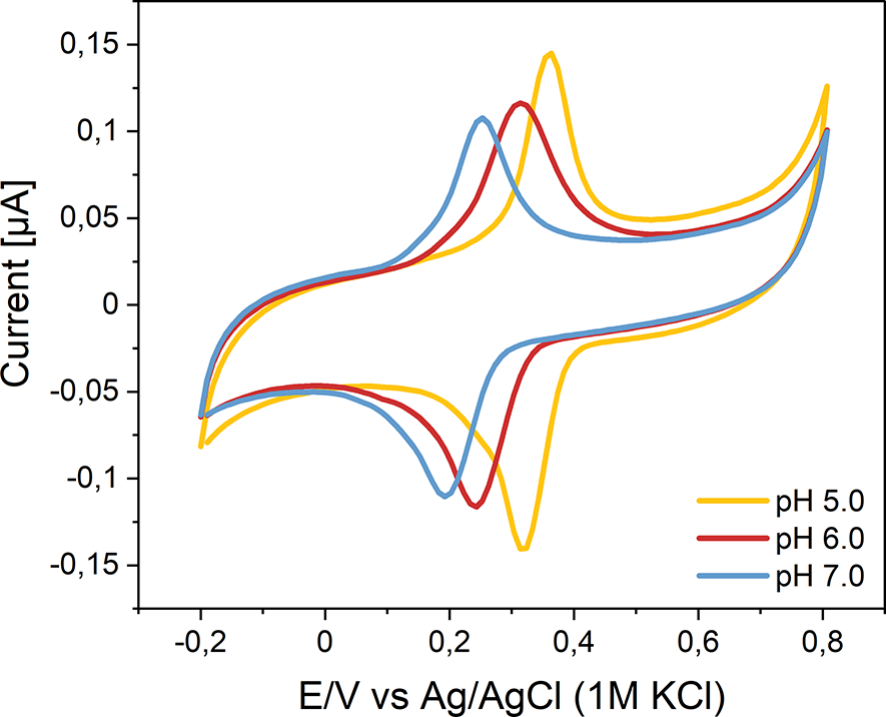
Selected CVs of pencil electrodes modified with syringaldazine recorded at various pH values of 0.1 M phosphate solutions.

Syringaldazine undergoes reversible one-step, two-proton, two-electron electrode reaction with a theoretical 59 mV change of the formal potential per pH unit. It easily adsorbs on carbon surfaces forming a stable layer allowing evaluation of solutions’ pH.^22^ However, the electrode modification easily deteriorates in a more complex medium (e.g., cell culture medium and real samples). Prolonged scans in buffer solution (Figure 2 left) and cell culture media (Figure 2 right) illustrate the effect of modification deterioration. In this work, syringaldazine was used as a model catalyst. We seek a surface modification that would not influence the electrochemical properties of the different sensors used in the future. If a given modification deteriorates the catalyst, influences the mechanism, or changes the environment of the reaction by, for example, a local change of pH, it should be discarded from future studies as it could lead to unexpected behavior of sensors and biosensors that we plan to use. The influence of the protective layer on the catalyst was evaluated through observation of the shape of voltammograms and the position of syringaldazine oxidation and reduction peaks depending on the pH (Figure 3).

The protective layer should be stable for at least 3 weeks, which is the growth time frame of dissociated neural cultures.^37^ For this reason, we evaluated the presence of the syringaldazine signal during periods of prolonged incubation in the cell culture medium.

### Protective Layers

Eleven different modifications were tested, including polymers forming porous, permselective membranes such as Nafion and polyphenylenediamine or polyvinyl chloride; hydrogels such as polyHEMA and PEGDE, silicate sol–gels, proteins, or carbon nanomaterial. The aim was to compare materials with the same setup of different antifouling mechanisms.

#### Polymers

Polymers form a physical barrier that protects from the access of species that could block the electrode surface. Their pores can provide permselectivity, excluding the passage of molecules due to their size or charge. The attachment of unwanted species is also reduced due to the much weaker interaction of foulants with many polymers, especially superhydrophobic ones, in comparison with the electrode surface.

Nafion is a porous membrane made from a hydrophobic tetrafluoroethylene polymer lined with hydrophilic sulfonic acid groups. They provide a negative charge when the membrane is exposed to an aqueous electrolyte.^38^ Nafions’ antifouling properties are based on the creation of a physical barrier and electrostatic repulsion of negatively charged pollutants. Both reduction and oxidation peaks of syringaldazine, with pH-dependent positions, were observed in the case of sensors covered with the drop-casted layer. However, the signal was unstable, and the intensity lowered with each scan. Results for the dip-coated sensors were not reproducible; no pH-dependent peak shift was observed, and we assumed that syringaldazine from the electrode dissolves in the alcoholic polymer solution during coating.

Another popular polymer often used for coating the sensor’s surface used for in vivo measurements^39^ is polyphenylenediamine. It blocks the access of bigger molecules but has excellent permeability toward H_2_O_2_, O_2_, etc. A 10–35 nm-thick film can be easily electrodeposited on the electrode surface. The procedure was repeated multiple times on glassy carbon electrodes and pencil graphite electrodes, and the signal during electrodeposition was consistent with literature data.^25^ However, no signal from syringaldazine could be observed after the electrodeposition of the OPD, or the signal was barely visible, which can be attributed to a more favorable interaction of the phenylenediamine with the electrode surface and desorption of the redox probe during the polymerization.

Polyvinyl chloride forms a hydrophobic, highly plasticized membrane, which can influence the selectivity of the sensor due to the difference in hydrophobicity of detected species^40^ but also serves as a protective barrier against proteins. It was already shown that the pore size can influence the long-term stability of sensors, with smaller pores providing greater stability in blood.^41^ A significant reduction of the syringaldazine signal was observed after coating the electrode with PVC. The protective layer also negatively influenced peak separation.

PLLA is one of the most widely used biobased, biodegradable polyesters. It comes in high and low molar mass forms, the first characterized by poor adhesion due to cracking caused by a high degree of crystallinity.^42^

**Figure 4:**
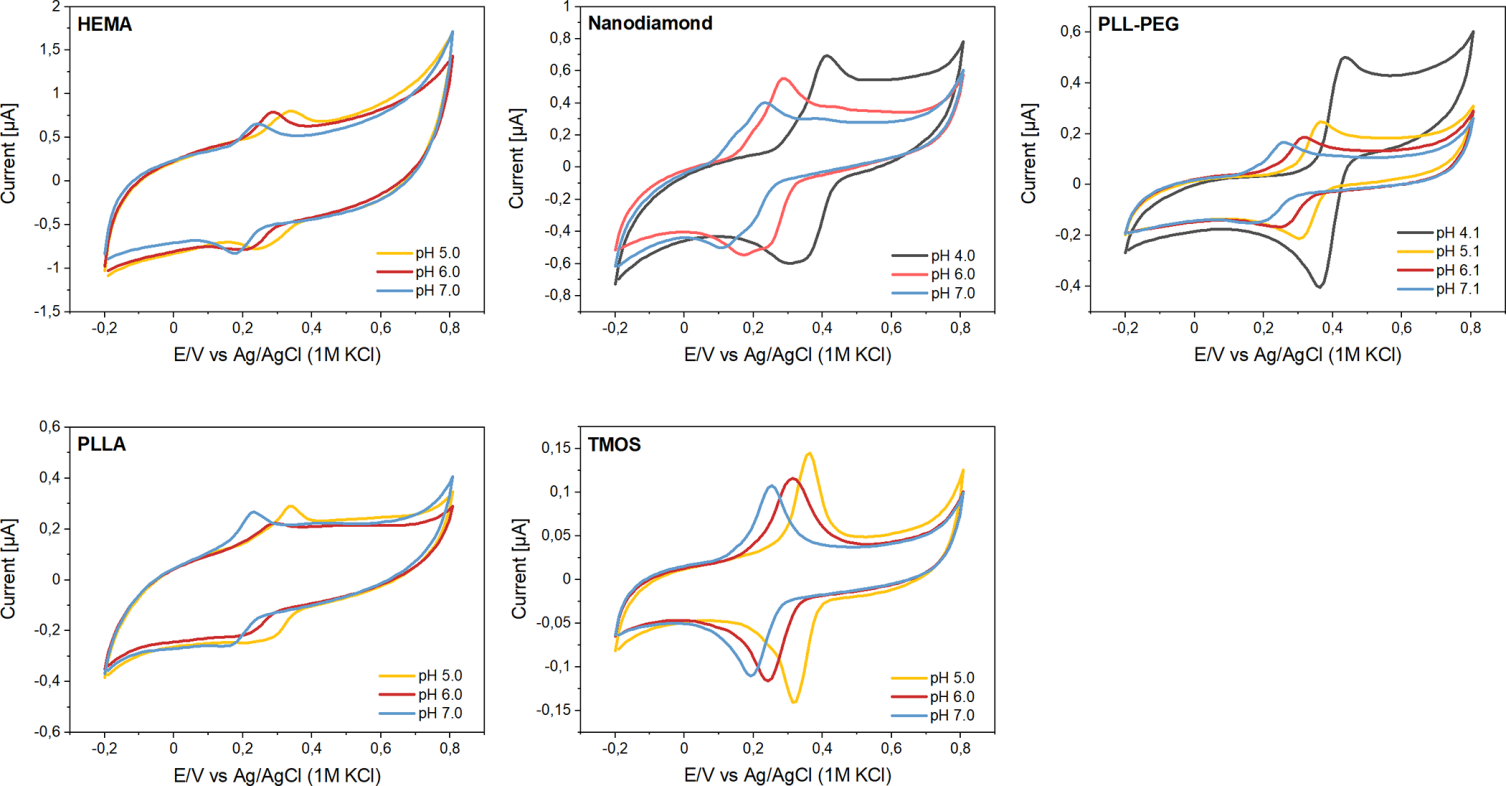
Cyclic voltammograms recorded at various pH values for syringaldazine-modified electrodes covered with selected protective layers: HEMA, nanodiamond, PLL–PEG, PLLA, and TMOS.

Therefore, low molar mass forms are preferred. The polymer is strongly hydrophobic^43^ and can therefore limit the adhesion of foulants. PLLA coating resulted in increased capacitance of the system, but well-developed peaks with a pH-dependent position (∼50 mV/dec) were clearly visible (Figure 4). Coating allowed the detection syringaldazine signals even after 3 weeks of incubation in a cell culture medium. PEG was shown to protect surfaces by hindering access to proteins, thanks to repulsive elastic forces. During compression, water molecules are removed from the hydrated polymer making penetration of other species thermodynamically unfavorable. However, this effect is highly dependent on the density of chains and their length and is usually not attainable for physical adsorption or covalent attachment. PEG is hydrophilic, flexible, and stable under sterilization but can suffer from autoxidation.^5,12,44^ PEG polymers can also be grafted with polypeptides, e.g., with poly(l-lysine), which is highly cationic at physiological pH, and the interaction with electrode surface can help to orient PEG chains by creating a well-structured polymer brush.^32^ Modification of electrodes with Poly(l-lysine)-*g*-Poly(ethylene glycol)[PLL–PEG] allowed observation of well-developed syringaldazine peaks (Figure 4) of pH-dependent position (50 mV/dec) throughout the whole course of the experiment in the cell culture medium.

Lack of compatibility between the organic catalyst, syringaldazine, and the polymer matrix can hinder the performance and stability in such mixed matrix membranes (MMMs). Polymers are only miscible with each other in the case of the absence of specific interaction for homopolymer-copolymer and homopolymer-homopolymer systems.^45^ The presence of the copolymer further increases the miscibility.^46,47^ The entropy of mixing per unit volume of small molecules as compared with polymers is large as the number of molecules involved in the process is greater.^48^ In the case of nanoparticles, their amount, radius, and localization all have an effect on the stabilization of the polymer system.^49^ In this study, the electrode is first coated with syringaldazine and later with the antifouling layer; thus, the catalyst does not form a mixture with the polymer. However, in the future, if the proposed biosensors should contain more components embedded in the polymer matrix, such thermodynamic effects should be taken into account.

#### Hydrogels

Hydrogels’ flexibility is similar to that of natural tissue. They are hydrophilic and uncharged. Their volume increases in aqueous solutions allowing analytes to easily diffuse in the swollen gel layer.^41^ Hydrogels are used extensively as scaffolds in tissue engineering and drug delivery systems. Their structure is often too weak to withstand implantation; for this reason, layers might benefit from additional cross-linking. Apart from physical barriers, hydrogels can also prevent fouling through strong repulsive hydration forces of the tightly bound water layer.^8^

PEG is a hydrophilic molecule used among others, to passivate surfaces and prevent nonspecific adsorption of proteins.^50^ As mentioned earlier, the ability to protect from fouling highly depends on the surface density and mutual orientation of PEG molecules.^5^ PEG can also be cross-linked to form a hydrogel with PEGDE frequently being the starting material.^51^ It is nontoxic, biocompatible, and stable under sterilization, and for this reason, it is often used to immobilize enzymes on implantable sensors.^28^ In the case of syringaldazine-modified electrodes, PEGDE increased the capacitance of the sensors, but both reduction and oxidation peaks were clearly visible. A slow decrease in the signal was observed with each subsequent scan, and no signal from syringaldazine could be detected after a weeklong incubation in the cell culture medium.

HMMA gels are transparent, chemically and thermally stable, with increased resistance to dehydration. PolyHEMA-coated sensors exhibited well-developed peaks of pH-dependent position, although an increase in capacitance was also notable (Figure 4). No deterioration of the signal was observed during repeated measurements in the buffer solutions. Prolonged incubation in cell culture medium resulted in a complete loss of signal, and no peaks could be observed after two week long incubation.

#### Sol–Gels

Silicate sol–gels are chemically inert, exhibit good mechanical and thermal stability, and are biocompatible and relatively low cost.^52^ They were shown to inhibit the settlement of zoospores.^53^ Due to high porosity, they are often used for enzyme immobilization for sensors and fuel cell applications. In this work, electrodes were coated with porous layers of silicate matrix formed as a result of the cross-linking of tetramethoxysilane (TMOS) (Figure 4) or tetramethoxysilane and TMA. The addition of the TMA cation results in higher porosity due to the presence of ordered defects.^54^ Initial results were similar for both layers; however, after just 1 day of incubation in a cell culture medium, a complete signal loss was observed for the TMA/TMOS modification. The TMOS modification peaks were significantly reduced but still observable after 3 weeks of incubation in the cell medium. The pH dependence was close to the theoretical value (∼60 mV/dec for reduction). It was one of the few modifications that withstood cell culture treatment during the entire time frame of the experiment.

#### Proteins

Although proteins are the main fouling agents, a common practice is to block the sensor surface with a homogeneous layer of a known protein. The surface is characterized after being blocked in a controlled manner, and it is assumed that further adsorption of species from the solution will change the signal only to a small extent.

Albumin is considered a biological fouling standard and is often used to test the electrode stability. As a potential antifouling agent, it was deposited directly and in this way did not present any protective properties. Electrodes in which albumin was mixed in a layer of PEGDE hydrogel were also prepared and characterized. The results in this case were analogous to the ones obtained with hydrogel; however, the layer was less stable, and fragments of the coating tended to detach during measurements.

#### Carbon Materials

The antifouling properties of some carbon materials are attributed to their high surface area and sp^3^ hybridization. A small number of polar functional groups on the surface of diamond particles greatly reduced the possibility of adsorption of numerous foulants. To attach the nanodiamond to the electrode, it was embedded in a previously prepared TMA/TMOS matrix. Observed results were analogous to pure silicate layer, and no enhancement due to the presence of nanodiamond was observed (Figure 4).

### Influence of Cell Culturing on Protective Layers

**Figure 5:**
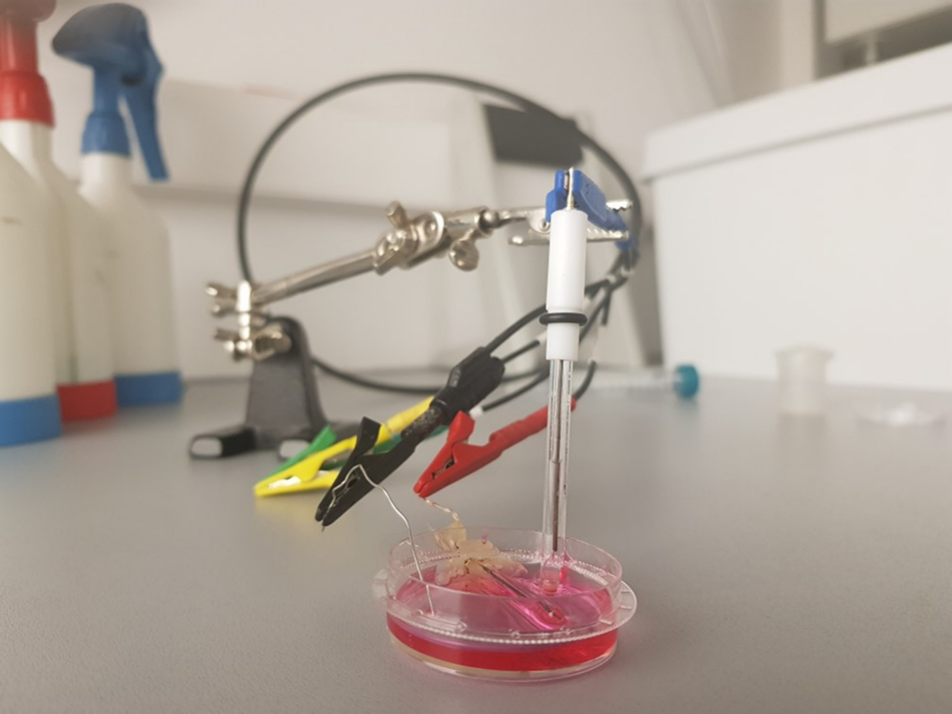
Photograph of the measurement setup, in which the working electrode was glued to the wall of the cell culture Petri dish. Reference and counter electrodes were positioned from the top for the time of the measurement.

Among all the compounds presented earlier, three of them turned out to be resilient to the cell culture medium and did not negatively influence the catalyst layer. Therefore, they were subjected to further examination this time in the cell culture. Working electrodes made from pencil graphite modified with syringaldazine and covered with the appropriate protective layer were immobilized in cell culture Petri dishes as shown in Figure 5. The working electrode was kept in the cell culture during the whole time frame of the experiment, whereas reference and counter electrodes were placed only during the measurements.

**Figure 6:**
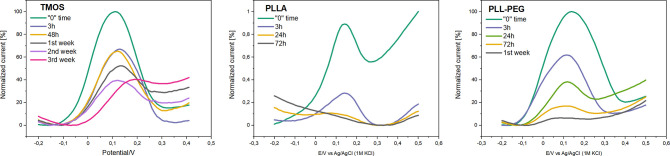
Deterioration of signal for TMOS, PLLA, and PLL PEG modifications during prolonged measurements with the electrodes immobilized in the cell culture dish.

Cell culture medium and especially metabolites excreted by the cells both have a huge impact on the antifouling layer. Comparison of electrode signal from different layers was based on DPV measurements and the heights of the individual peaks. A 100% height of the DPV peak was taken at the beginning of experiments, which means just after HeLa cells were seeded on the tissue culture dishes and medium culture was added. Signal loss during prolonged measurements was calculated by taking the average of the six repeats. As expected, major changes are observed during the first hours of contact with the cell culture. After 3 h, the peak area was reduced by half in the case of the TMOS layer or a quarter in the case of PLLA. The experiments showed that the electrodes modified with TMOS layer could endure in cell culture for up to 6 weeks, PLL–PEG for up to 2 weeks, and complete loss of syringaldazine signal was observed for PLLA after just 72 h (Figure 6).

### Cytotoxicity of Different Layers

We performed alamarBlue cytotoxicity assays to investigate whether compounds designed to be an antifouling layer have toxic effects on HeLa cells. This standard cellular assay allows for cell proliferation and cytotoxicity effects study. In the living cells, after intracellular uptake due to the response of cellular metabolic reduction, resazurin is reduced to the fluorescent resorufin, allowing for reading based on adsorbance or fluorescence.

Inverted fluorescent microscopy images for 24 h incubation revealed that, on the PLLA and PLL–PEG layer, HeLa cells were not properly attached.

Therefore, the very low number of cells calculated from the viability assay let us think that part of the cells, which were not attached to the well bottom, was washed out during medium change. However, it does not indicate a toxic effect of the layers, per se. It could be confirmed by microscopy images of the dishes with attached electrodes although the electrodes do not have direct contact with a dish bottom. For the TMOS layer, the survival of HeLa cells incubated was almost 50%, which was much higher than in other cases (Figures S2–S4). Considering the above, it can be assumed that neither of the tested layers is nontoxic to HeLa cells and can be used as an antifouling material in the cell culture medium.

## Conclusions

In this study, we have prepared and tested more than ten antifouling layers for electrochemical sensors based on different mechanisms of action. Layers included porous materials, permselective membranes, hydrogels, silicate sol–gels, use of proteins, and sp^3^ hybridized carbon. A redox mediator used for evaluation was adsorbed on the electrode surface, which allowed testing of not only the protective effect of the antifouling modification but also its impact on the catalyst itself. A characteristic that is often omitted in such studies. Commonly used layers such as Nafion, polyphenylenediamine, and PVC negatively impacted the syringaldazine signal. Such information was previously lacking as studies are usually performed with an external redox probe present solution.

We indicated which materials (TMOS, PLLA, PLL–PEG) were able to maintain, although significantly reduced, signal for prolonged incubation in cell culture medium and did not influence the performance of the tested catalyst. We noted considerable differences in the signal resilience in cell cultures between each of the tested layers. As expected, the first hours of incubation in cell culture were crucial for the performance of the electrodes. Although the signal from the electrodes modified with the TMOS layer was reduced by half within the first 3 h, the signal was still visible for up to 6 weeks. On the other hand, the PLLA modification was less impacted during the initial hours of study; however, after 72 h, a complete loss of signal was observed.

## Data Availability

Data is available at doi:10.18150/U1ZMSZ in the RepOD repository.

## Supporting Information Available

Selected CVs of glassy carbon and screen-printed electrodes modified with syringaldazine recorded at various pH values; comparison of HeLa cell proliferation on 96-well plate for different layers; HeLa cells proliferating on Petri dish next to electrode without modification; and viability of cells on the experimental layers after 24 h measured with alamarBlue cytotoxicity assays (PDF)
