# Supplementary material for: Methods of Protection of Electrochemical Sensors against Biofouling in Cell Culture Applications
Source: ACS Omega. 2024 Jan 18;9(4):4572–80. doi: 10.1021/acsomega.3c07660 (PMC10831843; doi:10.1021/acsomega.3c07660)
Supplement: Supplementary file 1 — ao3c07660_si_001.pdf [file ao3c07660_si_001.pdf]

Supporting Information: Methods of protection of electrochemical sensors against biofouling in cell culture applications

Elżbieta Jarosińska, Zuzanna Zambrowska1[1], Emilia Witkowska Nery*

Institute of Physical Chemistry, Polish Academy of Sciences, Warsaw, ul. Kasprzaka 44/52, 01-224 Warsaw, Poland *e-mail: ewitkowskanery@ichf.edu.pl, http://sensorarrays.com.pl/

KEYWORDS (Bio)fouling, electrodes, cell culture, tetramethoxysilane (TMOS) silicate matrix, pencil graphite electrodes

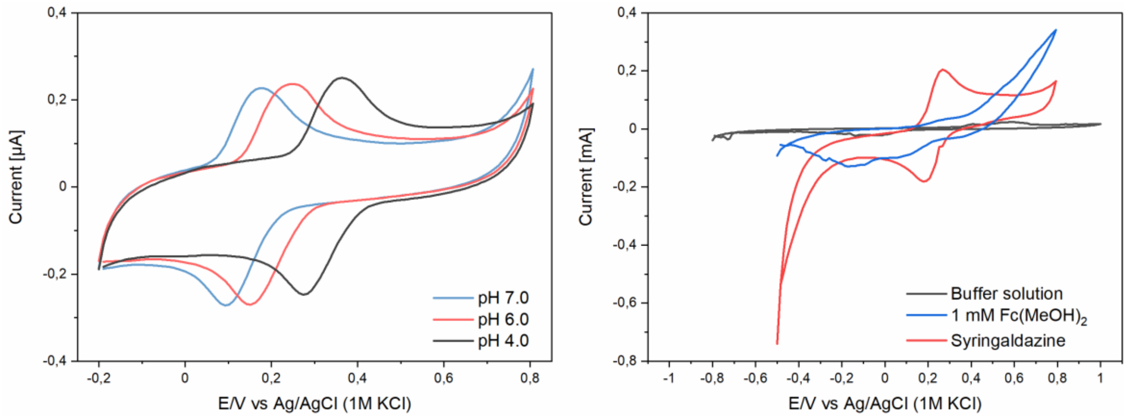

Figure SI1 Selected CVs of glassy carbon(left) and screen-printed electrodes(right) modified with syringaldazine recorded at various pH values of 0.1 M phosphate buffer solutions.

[1] Present address: Department of Clinical Physiology, Centrum Medyczne Kształcenia Podyplomowego, Warsaw, Poland

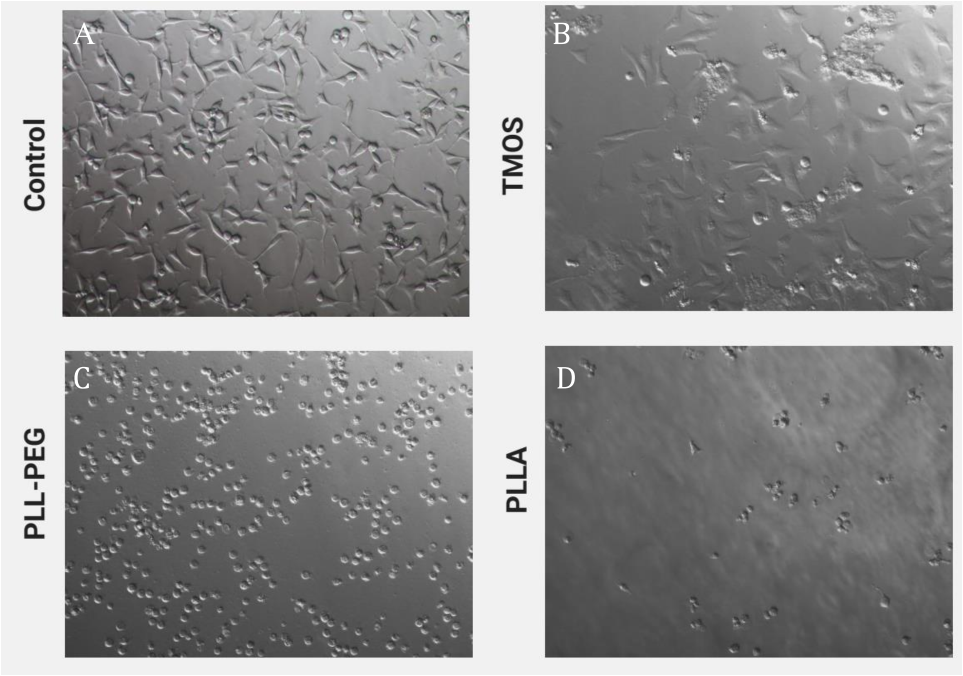

Figure SI2 Comparison of HeLa cells proliferation on 96 well plate: control (A), TMOS layer (B). PLL-PEG layer (C) and PLLA layer (D).

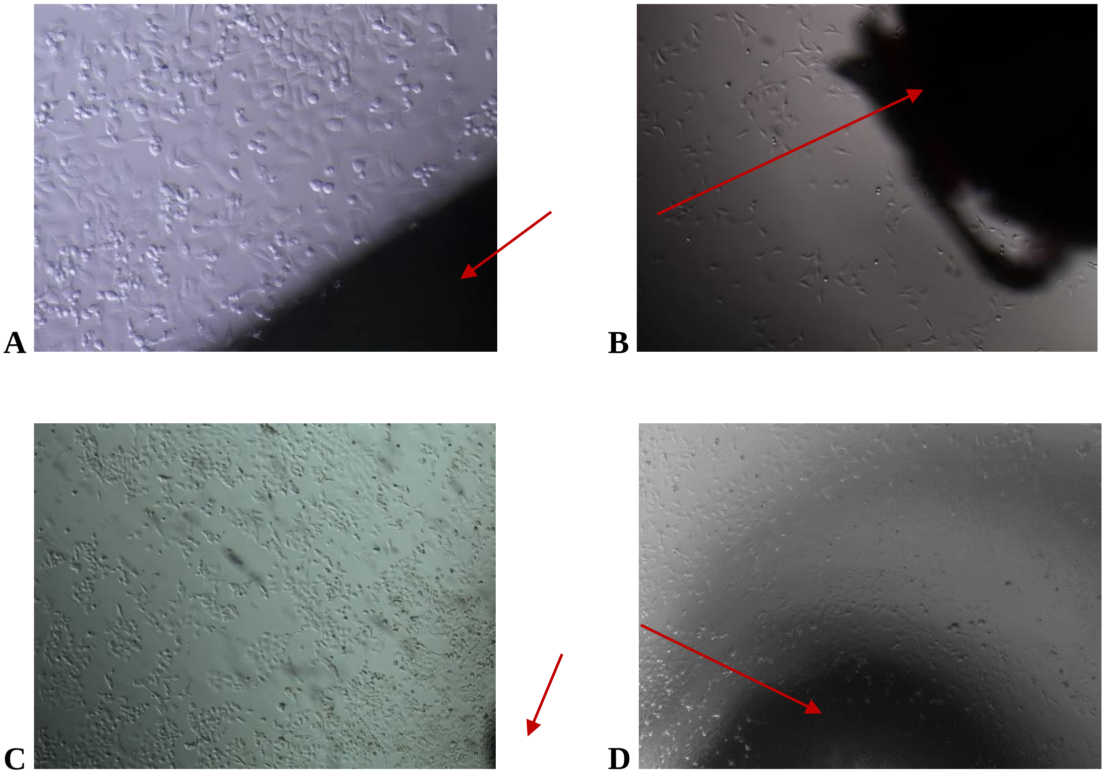

Figure SI3 HeLa cells proliferating on Petri Dish next to electrode without modification (A) and electrodes modified by PLLA (B), PLL-PEG (C) and TMOS (D). Electrodes were immersed in cell culture medium but above cells, therefore they stand out as a shadow in pictures (marked with red arrows).

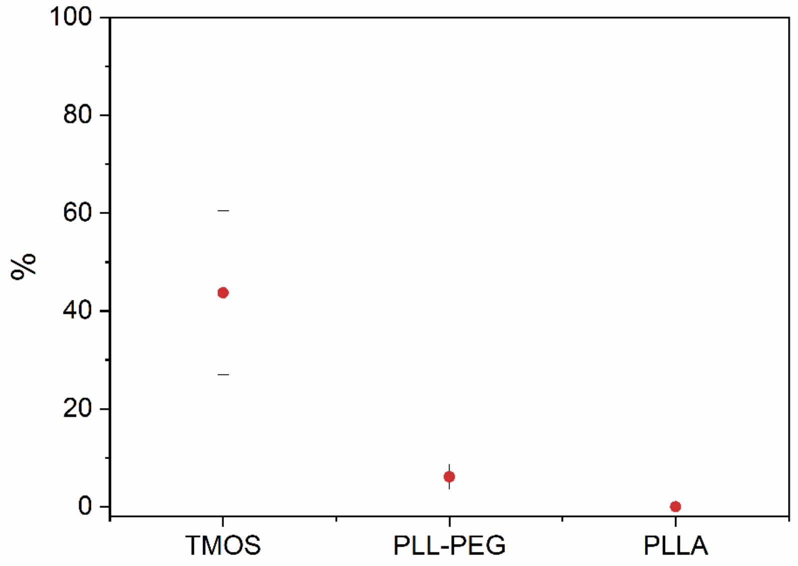

Figure SI4 Viability of cells on the experimental layers after 24h measured with alamarBlue cytotoxicity assays (st dev calculated for N=6).
